# Prophylactic Inoculation

**Published:** 1902-09-13

**Authors:** 


### The Hospital

A Journal of

The Medical Sciences and Hospital Administration.

Vol. XXXII.—No. 833. Edited by Sir Henry Burdett, K.C.B., and Solomon C. Smith, M.D., M.R.C.P. [September 13, 1902.

### PROPHYLACTIC INOCULATION.

The proposal of the Punjab Government to make a serious attempt to stem the course of plague by widespread inoculation, should that disease unfortunately become prevalent again during the coming cold months, is one which deserves the most careful consideration, and one, moreover, which may well draw upon itself a certain degree of criticism, notwithstanding the fact that the proposal is founded upon responsible medical recommendations. The greatness of the evil which it is hoped thereby to stay is recognised by all. In the districts under consideration plague has during the past three winters increased in an alarming ratio. From 530 deaths in the cool season of 1899–1900 the mortality sprang up the next year to 6,399, while last winter 23 British districts and nine native States were attacked, and about 200,000 deaths were caused by the disease. Moreover, this sad mortality took place not in parts of the country in which the malady had been allowed to run its course without any efforts being made to hold it in check, but in districts where well-directed efforts appear to have been made to prevent its spread. Extensive measures for evacuating the affected houses have been put in force, disinfection is said to have been carefully tried, and it is in consequence of the failure of these proceedings and the apparent ineffectiveness of ordinary sanitary measures, and in view of the prospect before them of another appalling outbreak, that the authorities are proposing this great scheme for inoculating about six and a half millions of people between now and next January. The area which is to be covered by this great experiment includes 14 considerable towns and a large rural district having a population of about ten millions, of whom it is hoped to inoculate two-thirds.

For the purposes of this measure a large force of English medical officers is to be engaged, no native officers under the rank of assistant-surgeons being employed in the work. Good pay is offered, and as the climate of that part of India is very pleasant during the winter months no difficulty in obtaining the men is anticipated. Of course the expense will be very heavy, but far less—at least so it is said—than even the mere money loss of an epidemic like that of last winter.

That this is an experiment which from a scientific point of view is worth trying, if the people are willing to try it, goes without saying, as also it does that if tried it should be carried out with all care in order that the knowledge to be gained therefrom may not be invalidated by any imperfection in the methods employed. It is, we fear, a policy of despair; but if done, let it be well done.

We cannot, however, fail to see that although the Punjab experiment stands far ahead of all others of a similar description, it does but mark and emphasise a drift in modern medicine which at the present time is making great headway. At the very moment that preventive inoculation for plague is brought into prominence by events in India, so is anti-typhoid inoculation by the publication of a general review of its results from the able pen of Professor Wright, of Netley, and one can hardly doubt that the reception given in scientific circles, and among people of influence, to these and other nearly related prophylactic measures, indicates a widespread tendency to regard inoculation as a ready way out of many difficulties in relation to public health. Yet if we turn aside from the individual instance, and consider how far it is desirable, or even justifiable, to protect large masses of people from the consequences of their sanitary shortcomings by inoculating them with the derivatives of disease germs we find ourselves embarked upon a very large and very doubtful question.

Vaccination against small-pox stands on a different footing. Not only is the process peculiar to itself, but so also is the certainty of its effect. Moreover, the virulence of the disease against which it is protective, and the readiness with which that disease attacks all sorts and conditions of men, practically irrespective of their surroundings, would drive us to accept vaccination even were it attended with far greater risks than in fact it is.

But the proposal to use inoculation as a means of obtaining protection against enteric fever, a disease the connection of which with sanitary defects is well recognised, is quite another affair, as also is the proposal to obtain immunity in the same way from such a disease as plague, one which while running riot among people whose habits encourage the access of the germ, hardly touches those who are able so to arrange the manner of their lives, the nature of their clothing, and the condition of their dwellings as to escape infection. To attack the evil consequences of insanitary living instead of meeting the difficulty by raising the standard of existence, is distinctly retrograde from an evolutionary point of view. We lay stress upon this point because there are at the present time indications of a considerable willingness to fly to these methods as short cuts to immunity, far easier to follow than the straight and narrow path on which sanitary progress has to plod along, and more especially because it has recently been stated that should this wholesale inoculation prove successful it will at once place this measure on a par with vaccination as the only practical method of dealing with a wide-spread epidemic manifestation of plague, a statement of far too serious a character to be passed over without comment.

